# Production of Hexaric Acids from Biomass

**DOI:** 10.3390/ijms20153660

**Published:** 2019-07-26

**Authors:** Riku Sakuta, Nobuhumi Nakamura

**Affiliations:** Department of Biotechnology and Life Science, Tokyo University of Agriculture and Technology, 2-24-16 Nakacho, Koganei, Tokyo 184-8588, Japan

**Keywords:** aldaric acids, biorefinery, biofuel cell, bioprocess, biorefinery, carbohydrates, electrochemistry, green chemistry, oxidation, sustainable chemistry

## Abstract

Sugar acids obtained by aldohexose oxidation of both the terminal aldehyde group and the hydroxy group at the other end to carboxyl groups are called hexaric acids (i.e., six-carbon aldaric acids). Because hexaric acids have four secondary hydroxy groups that are stereochemically diverse and two carboxyl groups, various applications of these acids have been studied. Conventionally, hexaric acids have been produced mainly by nitric acid oxidation of aldohexose, but full-scale commercialization has not been realized; there are many problems regarding yield, safety, environmental burden, etc. In recent years, therefore, improvements in hexaric acid production by nitric acid oxidation have been made, while new production methods, including biocatalytic methods, are actively being studied. In this paper, we summarize these production methods in addition to research on the application of hexaric acids.

## 1. Introduction

**Figure 1:**
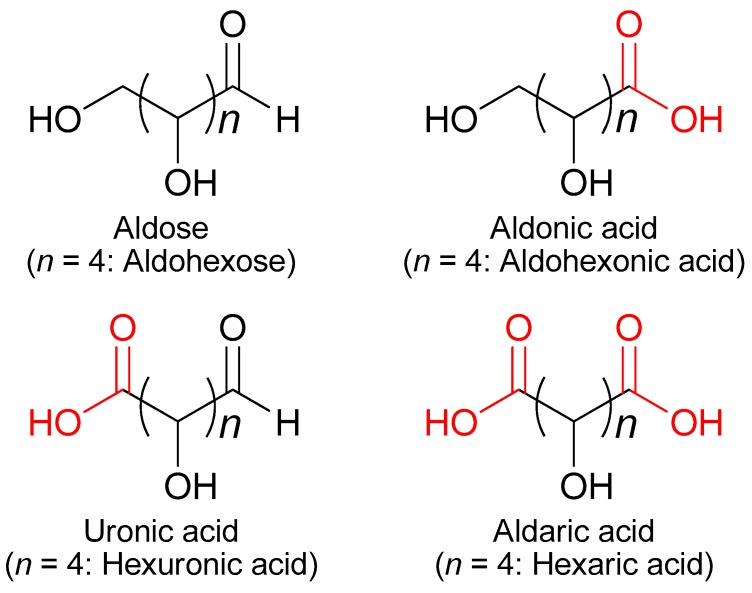
Structures of aldose and its acids.

The International Energy Agency defines a biorefinery as “the sustainable processing of biomass into a spectrum of marketable products and energy”, which is the most comprehensive and commonly accepted definition [1]. Because inexpensive petroleum-derived chemicals are already mass produced, the production of bio-based chemicals has been limited to those with structures that are too complex for the fine-chemicals market to justify their expensive production costs [2]. However, nonrenewable resources are limited despite population growth. Accordingly, the increased consumer demand for environmentally friendly products has become a driving force for the use of biorefineries [2]. Emerging economies (e.g., the countries of Brazil, Russia, India and China (BRIC)) require increasing amounts of oil and other fossil-based products, in addition to the security of chemical and energy supplies for isolated regions such as islands [2]. Carboxylic acids have attracted considerable attention among the raw materials for bioderived chemicals. The US Department of Energy (DOE) has selected twelve chemicals from more than three hundred biomass-derived chemicals, based on cooperative research with industry and academia, to be developed using biorefinery production methods [3]. More than half of the twelve selected chemicals are carboxylic acids. Carboxylic acids obtained by the oxidation of monosaccharides and oligosaccharides are referred to as sugar acids [4,5,6]. The oxidation of aldose (aldohexose when the carbon number is six) at its aldehyde group to form a carboxyl group produces aldonic acid (aldohexonic acid), whereas the corresponding oxidation at its terminal hydroxy group results in a different monocarboxylic acid, (aldo)uronic acid ((aldo)hexuronic acid). Aldaric acid (hexaric acid) is a dicarboxylic acid produced by oxidizing both groups (Figure 1). Because aldaric acids have been studied for numerous types of applications, improved methods for their production are urgently needed. d-Glucaric acid, the aldaric acid of d-glucose or l-gulose, was selected by the DOE as one of the twelve chemicals [3]. Hence, this article summarizes biorefinery methods relating to aldaric acids with six carbon atoms (hexaric acids; Figure 1), including d-glucaric acid.

## 2. Classification of Aldohexoses and Hexaric Acids

**Figure 2:**
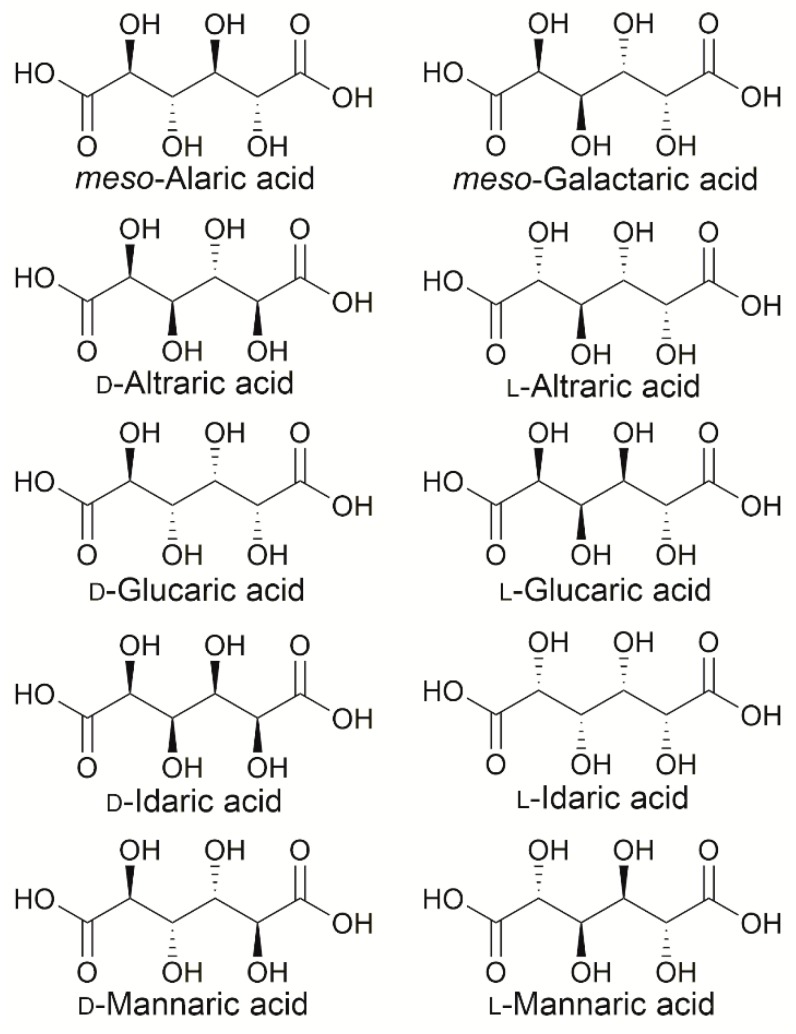
Structures of hexaric acids.

Aldohexoses have four stereocenters, resulting in sixteen configurational isomers (Figure 1). Relative-configuration classification divides these stereoisomers into eight groups with the following common names: altrose, allose, idose, galactose, glucose, gulose, talose, and mannose. In contrast, hexaric acids have the same functional group at both ends of their structure; thus, the hexaric acids of altrose and talose and those of glucose and gulose are the same compounds. Moreover, the hexaric acids of allose and galactose are *meso*-compounds, because of their symmetry. In short, relative-configuration classification divides hexaric acids into altraric acid, allaric acid, idaric acid, galactaric acid, glucaric acid, and mannaric acid; because allaric acid and galactaric acid are *meso*-compounds, the total number of configurational isomers is ten (Figure 2) [7]. Because hexaric acids have four stereochemically diverse secondary hydroxy groups and two carboxyl groups, their applications as platform chemicals have been studied as described below. Of the hexaric acids, d-glucaric, *meso*-galactaric, and d-mannaric acids have been the most studied as starting compounds for biorefineries, in part because the raw materials for these hexaric acids are abundant. Therefore, several compounds, such as l-mannaric acid [8,9] and d-idaric acid, [10,11] which are used as the starting compounds for human immunodeficiency virus (HIV) protease inhibitors, are regarded as exceptions in this report. The focus of this article is on studies investigating the applications and production methods for d-glucaric acid, *meso*-galactaric acid, and d-mannaric acid.

## 3. Applications of Hexaric Acids

Because hexaric acids have four stereochemically diverse secondary hydroxy groups and two carboxyl groups, they have been studied since the 1950s as platform chemicals for producing chelating agents and corrosion inhibitors [10,11,12,13,14,15,16,17,18,19,20,21], precursors for polyamides [22,23,24,25,26,27,28,29,30,31,32,33,34,35,36], polyesters [37,38,39,40,41], polyanhydrides [42], polycations [43], coordination polymers including metal–organic frameworks [44,45,46,47,48], pendant polymers [49], macromolecules [50,51], cross-linkers in hydrogels [52], medicines [8,9,10,11,49,51,53], and other compounds including platform chemicals, like adipic acid and furan dicarboxylic acid [54,55,56,57,58,59,60,61,62]. 

### 3.1. Monomers

**Figure 3:**
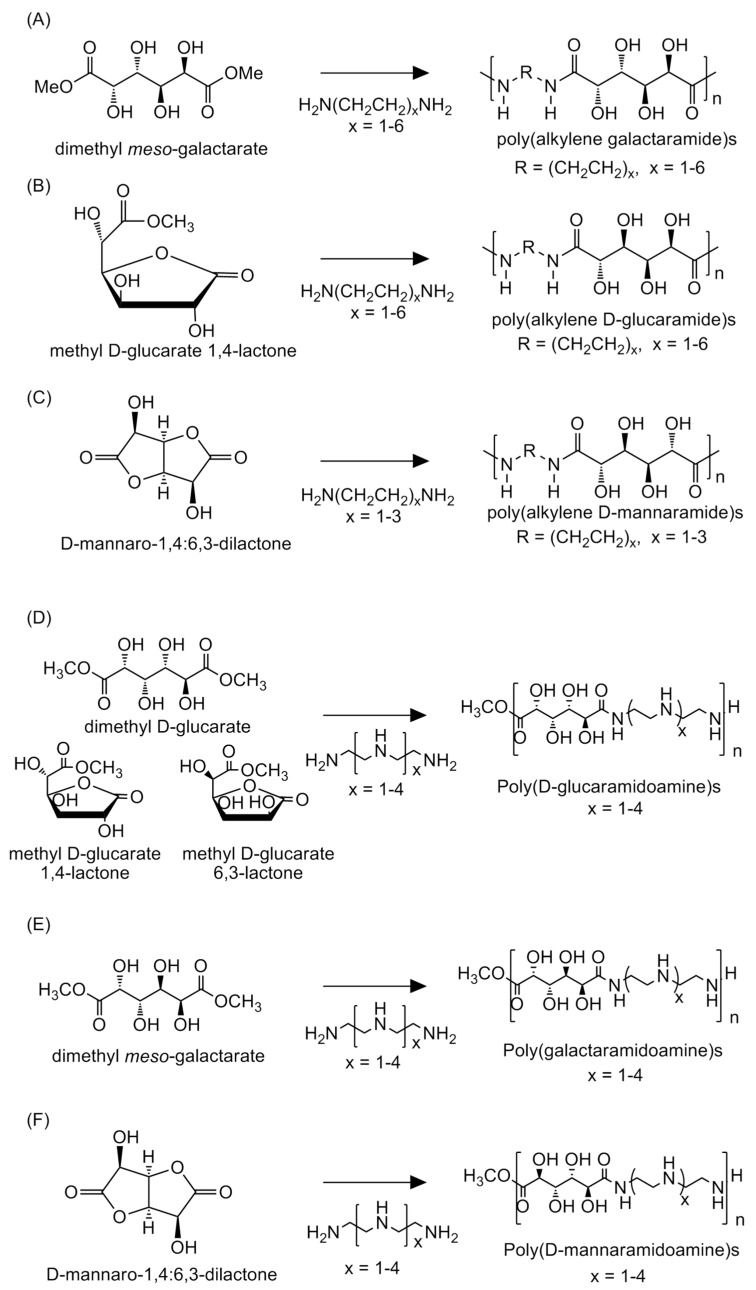
Schematic depiction of the syntheses of poly(alkylene galactaramide)s (**A**), poly(alkylene d-glucaramide)s (**B**), and poly(alkylene d-mannaramide)s (**C**) from their corresponding monomers and alkylenediamines [31] and the syntheses of poly(d-gulcaramidoamine)s (**D**), poly(galactaramidoamine)s (**E**), and poly(d-mannaramidoamine)s (**F**) from their corresponding monomers and secondary amines [35].

The use of hexaric acids as monomers produces polycondensates that are nontoxic, biodegradable, and more hydrophilic than those derived from petrochemicals [36]. Research on polyamide syntheses derived from hexaric acids started in the 1950s [22] and continues, particularly in the laboratory of Kiely. This group has synthesized polyamides from three monomers, d-glucaric acid, *meso*-galactaric acid, and d-mannaric acid, to investigate the influence of stereochemistry on the physical properties of polymers [31]. The polyamides were synthesized in methanol from either a hexaric acid diester or a methylated hexaric acid lactone, which were synthesized by a condensation reaction of a hexaric acid and methanol, with even-number-chain-length alkylenediamines having two to twelve carbon atoms as the monomers (Figure 3A–C).

A comparison between poly(alkylene galactaramide)s synthesized from *meso*-galactaric acid and poly(alkylene d-glucaramide)s from d-glucaric acid revealed that the melting points of poly(alkylene galactaramide)s were all higher than those of poly(alkylene d-glucaramide)s when the length of the alkylenediamine used was the same. Furthermore, it was found that, while the poly(alkylene d-glucaramide)s composed of alkylenediamines with two and four carbon atoms were water soluble, only the poly(alkylene galactaramide) produced from ethylenediamine was water soluble (Table 1). *meso*-Galactaric acid has a highly symmetric structure in which the carbon chain extends in a zigzag, bearing alternating hydroxy groups as confirmed by crystallography [63]. Through these hydroxy groups, *meso*-galactaric acids can interact via intermolecular hydrogen bonding [63]. In the case of d-glucaric acid, which is asymmetric, the 1,3-steric repulsions between the hydroxy groups at the C2 and C4 positions result in a bent molecular structure rather than an extended-zigzag structure [64]. Kiely et al. reported that the stereochemical differences between hexaric acids were responsible for the differences in the physical properties between the polyamides [31]. Because the galactaric acid moiety is extended, poly(alkylene galactaramide)s readily become linear. The adoption of a linear structure could increase both the hydrogen bonding between the galactaric acid moieties and the van der Waals forces derived from alignment of the alkylenediamine moieties. In contrast, poly(alkylene d-glucaramide)s tend to form a bent structure because the d-glucaric acid moiety is bent, preventing attractive forces from developing between the polymers. The authors concluded that the differences in interchain attraction underlay the differences in melting points and water solubility between these compounds. Poly(alkylene d-mannaramide)s have head-to-tail type stereoregularity because of the hydroxy groups bound symmetrically along the carbon chain of d-mannaric acid moiety. Because the 1,3-steric repulsions are not present in d-mannaric acid, the authors assumed that the acid tends to form extended-zigzag structures, resulting in melting points and water solubility similar to those of poly(alkylene galactaramide)s. Polymer syntheses that take advantage of physical property differences derived from the different stereochemical properties of aldaric acids have been reported. For example, a polymer derived from d-glucaric acid has an amorphous nature because the attractive interactions between the polymers are weak, which is advantageous for synthesizing materials that have good film-forming and adhesive properties [30]. The nature of *meso*-galactaric acid-derived polymers, which tend to form linear structures, was used to improve the thermophysical properties of polyanhydrides [42]. Henkensmeier et al. reported that they were able to change the physical properties of a polymer by combining d-glucaric acid and *meso*-galactaric acid; their method allowed for precisely adjusting the glass transition temperatures of silicone surfactants [34].

### 3.2. Application of Hexaric Acid Polymers: An Example

Applications of synthetic polymers, including as drug delivery carriers, have been reported. Liu and Reineke et al. reported polyamides composed of hexaric acid moieties and secondary amines as the repeating units that could replace viruses as nucleic acid medicine carriers (Figure 3E–G) [35]. Conventionally, polyethylene imines (PEIs) and chitosan have been studied as substitutes for viruses. The PEIs used in this study showed high gene-transport efficiencies in almost all cell lines used in the study, and chitosan displayed no cytotoxicity. However, each polymer also had a drawback: longer PEIs showed strong cytotoxicities, and chitosan had an inferior transport efficiency. The synthesized polyamides in this study had an abundance of secondary amines and hydroxy groups, and showed both good biocompatibilities and high transport efficiencies. Similar to PEI and chitosan, the synthesized polyamides formed viral-like electrostatic complexes with plasmid DNA (pDNA), termed polyplexes, which were taken up through the endocytotic pathway and dissociated within the endosome. The stereochemistry between the hexaric acid moiety and the number of amines in the repeating units greatly changed the binding affinity to pDNA, its volume in the binding complex, and its transportation efficiency into cells. The polymers derived from *meso*-galactaric acid showed higher transport efficiencies than those derived from d-glucaric acid or d-mannaric acid.

### 3.3. Macromolecules

**Figure 4:**
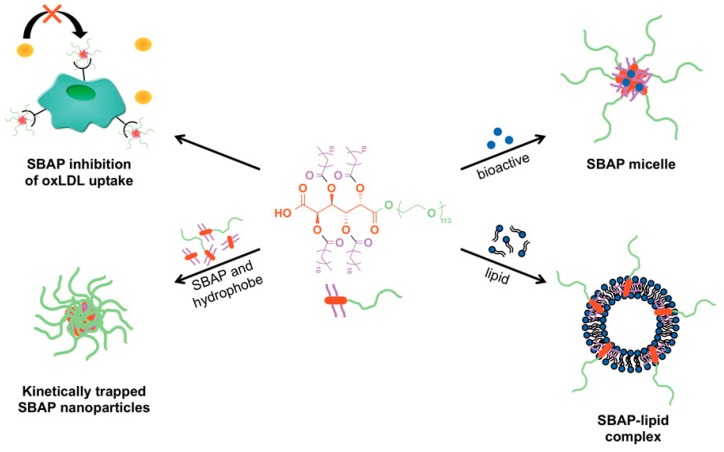
Sugar-based amphiphilic polymers (SBAPs) for biomedical applications [51]. oxLDL = oxidized low-density lipoproteins.

Macromolecules derived from hexaric acids have also been studied for use in drug delivery systems [50,51]. Gu et al. synthesized amphiphilic macromolecules by bonding the hydroxy groups and a terminal carboxylic group of the hexaric acids, commencing with *meso*-galactaric acid, to alkyl chains and poly(ethylene glycol)s, respectively (Figure 4) [51]. 

The resulting amphiphilic macromolecules, which showed low critical micelle concentrations, were studied for use as drug carriers for hydrophobic anticancer agents and gene therapy agents. Furthermore, these amphiphilic macromolecules were found to treat atherosclerosis by preventing macrophage intake of oxidized low-density lipoproteins.

### 3.4. Chelating Agents, Coordination Polymers and Metal–Organic Frameworks

Complexation of the carboxyl and hydroxy groups of hexaric acids with metals has been used in applied research. For example, such products have been investigated for use as agents to remove metals from polluted environments and incinerator plants, as corrosion inhibitors, and as Ca-sequestering agents, several of which have been commercialized [6]. The chelation ability of hexaric acids was exploited for the synthesis of coordination polymers and metal–organic frameworks (MOFs). Abrahams et al. synthesized coordination polymers derived from hexaric acids and metals. For example, they used d-glucaric acid and Zn to form a coordination polymer with two types of isolated, parallel channels, one of which was hydrophilic and the other hydrophobic [44]. The hydroxy groups of d-glucaric acid were exposed in the hydrophilic channels, while the hydrophobic channels had C-H bonds along the channel wall. Water molecules were in both channels, especially in the hydrophilic channel where they were hydrogen bonded. The water molecules in the hydrophobic channels were easily exchanged for azobenzene, I_2_, elemental sulfur, hydrocarbons, CCl_4_ and CI_4_. This material was expected to be applicable to the simultaneous introduction of two different species into the different channels.

**Figure 5:**
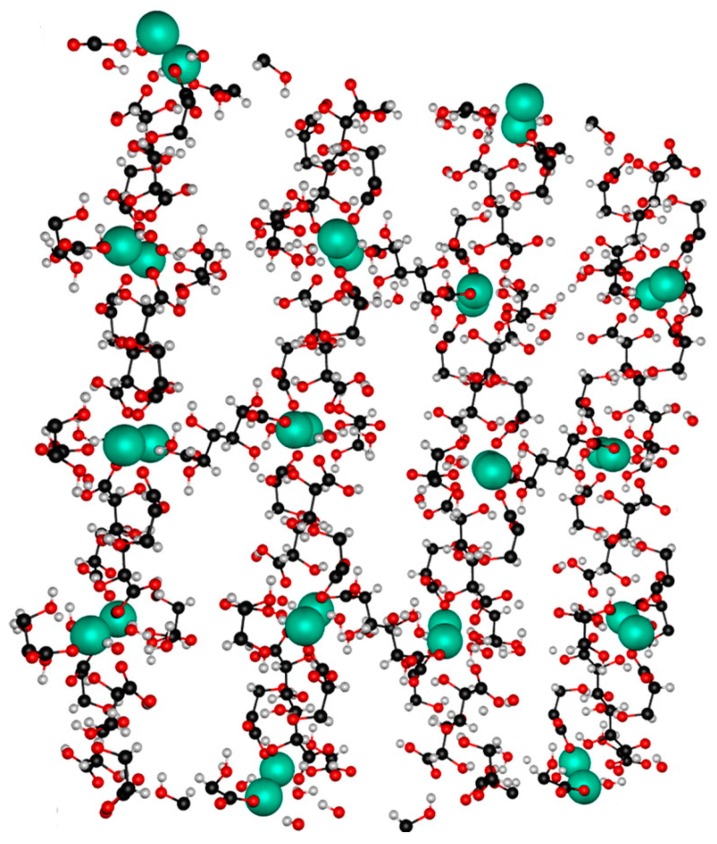
Crystal structure of the neutral metal–organic framework (MOF) (Tb(galactarate)_1.5_(H_2_O)_2_)·5H_2_O shown in a ball-and-stick representation, with the Tb(III) ions in a space-filling model. Water molecules in the channels have been omitted for clarity, and atoms are color coded as follows: C = black; H = white; O = red; Tb = turquoise [48].

Wong et al. synthesized a porous structure from *meso*-galactaric acid and terbium that could detect guest molecules (Figure 5) [46]. This structure formed a two-dimensional networked complex that created vertical one-dimensional channels when stacked in layers. Crystallography of this MOF revealed that the layers were connected through hydrogen bonding via water molecules; however, heating removed the water molecules without destroying the structure. In aqueous solutions, this MOF could remove I^−^, Br^−^, Cl^−^, F^−^, CN^−^, and CO_3_^2−^ and small amounts of SO_4_^2−^ and PO_4_^3−^. These anions were assumed to be removed by forming hydrogen bonds with the *meso*-galactaric acid moieties. The fluorescent properties of terbium, a lanthanide element, were successfully utilized to monitor the intake of anions into the MOF. Desorption of the anions from the MOF was confirmed by placing the absorbed complexes in pure water. 

### 3.5. Other Platform Chemicals

Hexaric acids have also been studied for use as the starting materials for other platform chemicals [54,55,56,57,58,59,60,61,62], including adipic acid [59,60,61,62], furan dicarboxylic acid (an alternative to terephthalic acid) [57], and pyrrole [55].

Li et al. reported the highly efficient conversion of *meso*-galactaric acid, via muconic acid, into adipic acid through an oxorhenium-complex-catalyzed deoxydehydration (DODH) reaction and following a Pt/C-catalyzed transfer hydrogenation [59]. Ionic liquid was integrated as a reaction medium into this DODH reaction in another paper [61]. The use of ionic liquid enabled an efficient separation of muconic acid by simple decantation. The recovered ionic liquid, which contained an expensive Re catalyst, was used up to four times without much of a decrease in yields. d-Glucaric acid can also be a starting compound for adipic acid. Recently, a catalyst system that provided adipate esters from d-glucaric acid in a single operation was reported, in which hydrogen was used as a terminal reductant [62]. 

According to the report by Thiyagarajan et al. [58], although the first report of furan-2,5-dicarboxylic acid from *meso*-galactaric acid was published in 1876, little attention has been paid to this route because isolated yields were moderate and *meso*-galactaric acid was not readily available. In terms of the availability of hexaric acids including *meso*-galactaric acid, it will be improved by the research mentioned in following sections. As for synthesis of furan-2,5-dicarboxylic acid from *meso*-galactaric acid, some works were reported. For example, one-step synthesis of dibutyl furandicarboxylates from *meso*-galactaric acid and 1-butanol, in the presence of sulfuric acid, was reported [57]. In this paper, the diester of furan-2,5-dicarboxylic acid was synthesized because it can be distilled or recrystallized by the use of existing solvents and can be polymerized directly using diols.

## 4. Hexaric Acid Production Using Inorganic Catalysts

**Figure 6:**
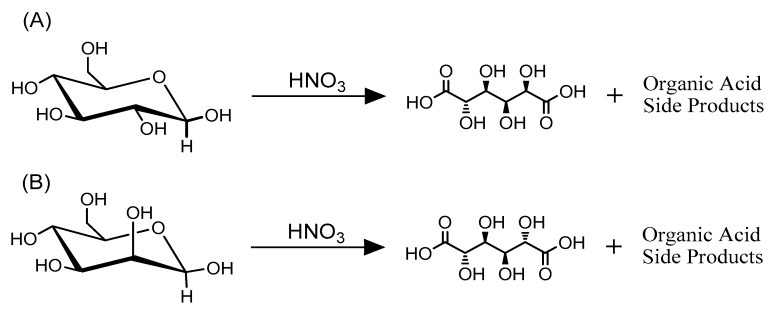
The nitric oxidation of (**A**) d-glucose and (**B**) d-mannose [67,69].

The earliest attempts of hexaric acid preparation were reported in the late 1800s (oxidation of carbohydrates with HNO_3_, of which the reaction origin could be traced back to lactose oxidation by Liebig and is still used today [65,66]). However, the yields using this method are limited to approximately 40% because of side reactions. Moreover, this method results in the excessive production of nitrogen oxide gas because of the rapid and highly exothermic nature of the oxidation reaction. These drawbacks have prevented the mass production of hexaric acids using this method [67,68]. Kiely’s group have improved d-glucaric and d-mannaric acid production via nitric acid oxidation to solve these problems, enabling industrial mass production of these hexaric acids (Figure 6) [67,69]. 

**Figure 7:**
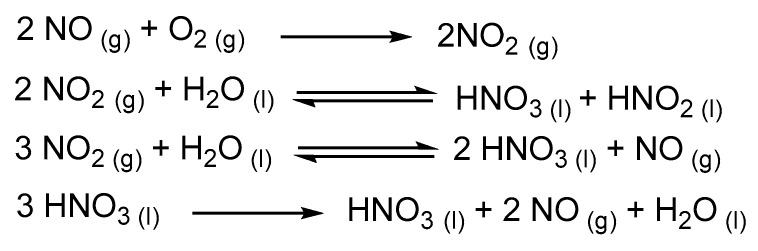
Common NO and NO_2_ reactions in water/O_2_ proposed in a previous report [69].

In their studies, the amount, rate, and temperature of the d-glucose and d-mannose additions were controlled by a computer to prevent rapid temperature increases caused by the oxidation of the saccharides by nitric acid and the resulting generation of large amounts of nitrogen dioxide and other gases. Relatively low reaction temperatures (25–40 °C) were accomplished. Furthermore, a positive oxygen pressure on the reaction system generated nitric dioxide from nitric monoxide, and the dissolution of the dioxide into water reproduced nitric acid, enabling reproduction and recycling of the nitric acid (Figure 7). The removal of nitric acid from the reaction mixture was also improved. 

To date, nitric acid has been removed by evaporation by heating under reduced pressure. However, complete removal is difficult because nitric acid and water form a negative azeotrope. Moreover, the relatively severe conditions used in this method allow for further oxidation reactions to occur in an uncontrolled manner. Therefore, nitric acid removal by nanofiltration and diffusion dialysis was also investigated in Kiely’s study. Selective nitric acid removal was achieved by both methods. However, because the addition of a large amount of water and pH adjustment was necessary, nanofiltration was not practical. Diffusion dialysis required neither the addition of water, the adjustment of pH, nor large amounts of energy for nitric acid removal. Further recycling of the removed nitric acid was easy. However, a problem remains in that the removed nitric acid solution contains a large amount of the products. Moreover, the yield of the reaction was not improved, remaining at 45% for d-glucaric acid. In addition to nitric acid oxidation, the production of hexaric acids by oxidation with bromine and manganese(III) sulfate has been reported [70,71,72,73]. Recently, ozonic oxidation has been proposed to solve several of the problems of conventional conversions, including their high environmental burden, byproduct generation, slow reaction rates, and high costs [74,75]. 

In addition to the use of strong oxidants, catalytic oxidation with transition metals has been explored. The Pt-catalyzed production of a hexaric acid, d-mannaric acid, was reported in 1938 [76]. Production of hexaric acids catalyzed by metals has been of industrial interest since the 1940s [77,78,79,80,81]. Following these antecedent works, several studies have been reported [68,82,83,84,85,86,87,88,89,90,91,92,93,94,95,96,97,98]. Although Pt [76,82,83,84,91,92,97] and Au [68,84,85,86,89,94,95,96] have been used as catalysts frequently, other elements like Ti [87,88], Mn [90], Fe [98], Mo [93], and Pd [91,99] can also be incorporated in catalyst composites. Aldohexoses [84,85,86,87,88,90,91,92,93,94,95,97,98], aldohexonic acids [82,83,84], and hexuronic acid [68,89,96], in addition to the corresponding sugar alcohol [76,85], can be starting compounds for the metal-catalyzed production of hexaric acids. In addition to the use of oxidants like H_2_O_2_ and O_2_, electrochemical [85,90] and photochemical [87,88] oxidation of the substrates has been examined. Recently, catalysis under autogenous pH conditions has attracted attention, although alkaline conditions were favored for some traditional efficient catalysis reactions [95,96,97].

Hexaric acid separation from the reaction mixture was based on various methodologies, including precipitation by lowering the pH and the use of ion exchange resins [67,69,100]. For example, d-glucarate was isolated by pH change (basification of the reaction mixture to pH 9.5, which was followed by acidification to pH 3–4 [67]). *meso*-Galactarate was also separated from the culture supernatant by acid precipitation, where 97% recovery was achieved with 97.5% purity at pH 6.5 [100]. d-Glucarate and d-mannarate were separated from each reaction mixture by ion exchange resins following their analyses [67,69].

## 5. Hexaric Acid Production Using Biocatalysts

Hexaric acid production using biocatalysts has also been studied. Biocatalysts, which are regenerable, enable environmentally friendly hexaric acid production. Furthermore, the substrate and reaction specificity of biocatalysts commonly omit the need for strict purification processes to extract the saccharides from the biomass [6].

Although a variety of dehydrogenases and oxidases are known to oxidize hexoses at the C1 position, few reports describe direct enzymatic oxidation of hexoses at the C6 position, such as eukaryotic and bacterial organisms that use uridine diphosphate (UDP)- and guanosine diphosphate (GDP)-bound hexoses as substrates to oxidize the C6 position. The products of these reactions are UDP- and GDP-uronic acids, which serve as the starting compounds for synthesizing polysaccharides, ascorbic acid, and other biomolecules [6,101,102,103,104,105].

**Figure 8:**
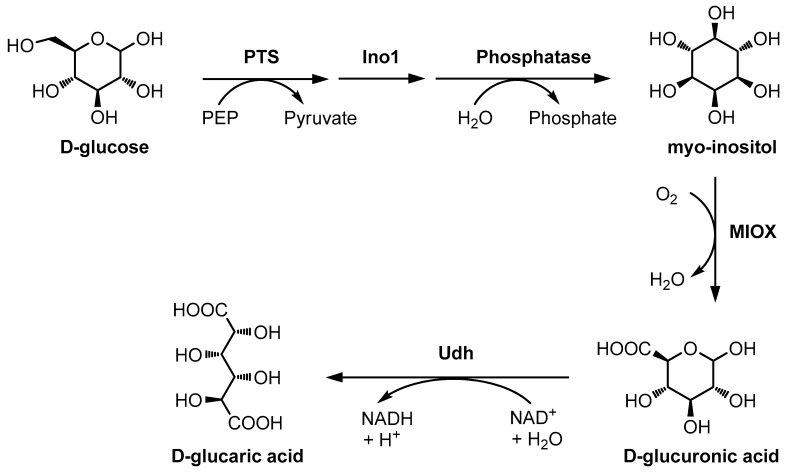
Designed pathway for the production of d-glucaric acid in *E. coli.* PTS = phosphoenolpyruvate-dependent phosphotransferase system; phosphatase = SuhB, an endogenous *E. coli* enzyme; PEP = phosphoenolpyruvate; MIOX = *myo*-inositol oxygenase; Udh = uronate dehydrogenase; [106].

Because direct oxidation of hexose at the C6 position is difficult, combinations of biocatalysts have been used to produce hexuronic acids. For instance, Moon et al. reported a method for the production of d-glucaric acid using genetically modified *Escherichia coli* (*E. coli,* Figure 8) [106]. In recombinant *E.* *coli*, phosphotransferase activity produces d-glucose-6-phosphate, which is then converted by *myo*-inositol-1-phosphate synthase into *myo*-inositol-1-phosphate. A phosphatase then converts the *myo*-inositol-1-phosphate into *myo*-inositol, which is then converted to d-glucuronic acid (the hexuronic acid of d-glucose) by *myo*-inositol oxidase. Moon’s report also describes the production of d-glucaric acid from the oxidation of d-glucuronic acid by nicotinamide adenine dinucleotide (NAD)-dependent uronate dehydrogenase (UDH). Because the yield of this reaction was low, improvements in the production system have been made [107,108,109,110,111,112]. Galactose oxidase is an uncommon enzyme that can oxidize hexoses at the C6 position. For example, d-galactose oxidation catalyzed by this enzyme produces d-galactohexodialdose [113,114]. The enzymatic oxidation of d-galactohexodialdose cannot produce hexuronic acid or hexaric acid because bicyclic structures are formed; however, the oxidation of dialdose by sodium chlorite is reported to produce d-galacturonic acid, the hexuronic acid of d-galactose [115]. Recently, a mutation in this enzyme, changing its structure, was reported to enable glucose oxidation at the C6 position [107,116,117].

As described above, UDP- and GDP-uronic acids have been utilized to synthesize polysaccharides in vivo; thus, hexuronic acids are abundant in nature as the main component of polysaccharides [6,101,102,103,104,105]. For example, d-galacturonic acid can be derived from pectin [118,119,120,121]. Alginic acid hydrolysis produces d-mannuronic acid and l-guluronic acid, the hexuronic acids of d-mannose and l-gulose, respectively [122,123,124,125,126,127]. d-Glucuronic acid can be obtained from hemicellulose [128,129]. d-Glucuronic acid can also be obtained as a monomer from ulvan [123,130]. Therefore, some studies have used hexuronic acids derived from polysaccharides, which are not used as food, as the starting compounds for hexaric acids.

**Figure 9:**
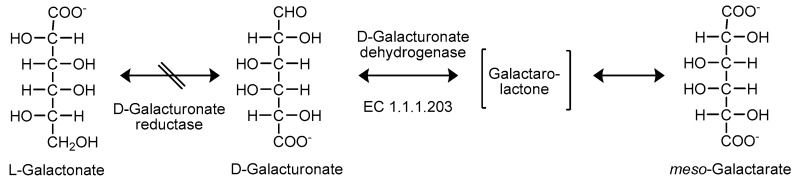
Engineering of the d-galacturonic acid pathway in fungi. Deletion of the gene encoding d-galacturonate reductase resulted in strains unable to utilize d-galacturonic acid as a carbon source. The expression of the bacterial *udh* gene, encoding an nicotinamide adenine dinucleotide (NAD)-dependent UDH (d-galacturonic acid dehydrogenase), resulted in fungal strains that were able to oxidize d-galacturonic acid to *meso*-galactaric acid. UDH forms a galactaro-lactone that spontaneously hydrolyzes [100].

Mojzita et al. reported the production of *meso*-galactaric acid from d-galacturonic acid using recombinant microorganisms in which the gene for UDH had been introduced [100]. Many microorganisms that live on putrid plants have enzymatic pathways that catabolize d-galacturonic acid. Eukaryotic microorganisms use a reduction pathway that starts with the reduction of d-galacturonic acid into l-galactonic acid, catalyzed by d-galacturonic acid reductase, while bacteria use an oxidative pathway that first produces *meso*-galactaric acid in a reaction catalyzed by UDH. In their study, Mojzita et al. eliminated the gene for d-galacturonic acid reductase in two eukaryotic microorganisms, *Aspergillus niger* (*A. niger*) and *Hypocrea jecorina* (*H. jecorina*), and introduced the gene for UDH, which was derived from the bacterium *Agrobacterium tumefaciens* (*A. tumefaciens*). Neither strain with a single-gene deletion of the reductase grew on medium in which d-galacturonic acid was the sole carbon source. In contrast, *meso*-galactaric acid production in both strains was confirmed when deletion of the reductase was accompanied by introduction of the dehydrogenase (Figure 9). Because the amount of *meso*-galactaric acid obtained was small in the case of *A. niger*, it is assumed that *A. niger* metabolizes *meso*-galactaric acid derived from d-galacturonic acid. Although a high yield of *meso*-galactaric acid was achieved with *H. jecorina*, the addition of d-glucose as an energy source was necessary because d-galacturonic acid cannot be used as the sole carbon source. Furthermore, the addition of d-glucose was found to decrease the yield of *meso*-galactaric acid. The fermentation method used to produce *meso*-galactaric acid has been improved [131,132,133,134,135,136]. 

**Figure 10:**
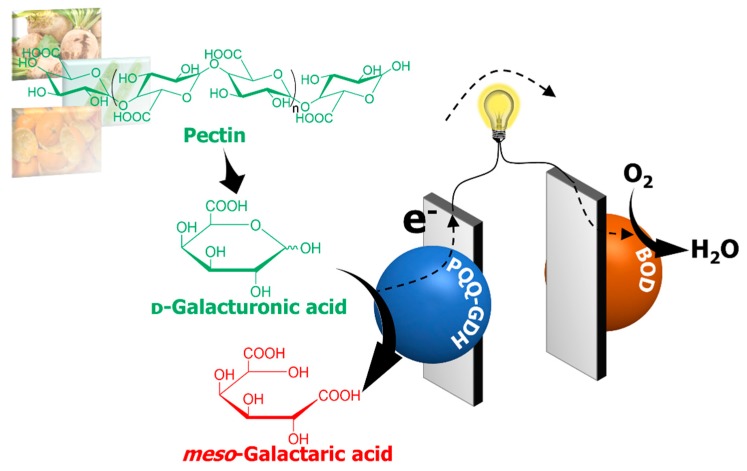
Schematic description of an enzymatic biofuel cell that simultaneously produces *meso*-galactaric acid and electricity from pectin extracted from biomass. PQQ-GHD = pyrroloquinoline quinone-dependent glucose dehydrogenase; BOD = bilirubin oxidase.

When chemicals are produced through an oxidative reaction, the electrons obtained from the substrate are usually transferred to electron accepters, such as O_2_. However, an enzymatic anode in a biofuel cell can serve as an electron acceptor. Enzymatic oxidative conversion on the anode can be combined with O_2_ reduction on the cathode to generate electricity. That is, the simultaneous production of chemicals and electricity is possible through oxidative conversion within enzymatic biofuel cells. This method reduces the energy required for producing chemicals and can even generate energy for other uses. The concept of a system that co-produces platform chemicals and electrical energy from biomass by demonstrating the simultaneous production of a hexaric acid and electrical energy using an enzymatic biofuel cell has been proposed [137,138,139]. The electrochemical oxidation of d-galacturonic acid and the production of *meso*-galactaric acid by a pyrroloquinoline quinone-dependent glucose dehydrogenase (PQQ-GDH)-modified electrode was confirmed [137,139]. The electrode was used as the anode in a biofuel cell with a bilirubin oxidase (BOD) cathode [139] (Figure 10). The cell generated electricity by using d-galacturonic acid and the hydrolysate of pectic acid as a fuel. This simultaneous production mechanism is applicable to other chemicals. We confirmed the oxidation of d-glucuronate and d-mannuronate, both catalyzed by PQQ-GDH, and the oxidation of l-guluronate, catalyzed by the PQQ domain of pyranose dehydrogenase from *Coprinopsis cinerea* (DH_PDH_) [138,139]. According to NMR measurements, the production of d-glucarate from the oxidation of d-glucuronate and l-guluronate and the production of d-mannarate from the d-mannuronate oxidation were confirmed. DH_PDH_ is also suitable for use as an anode catalyst. These combinations of enzymes and chemicals are applicable to simultaneous production systems. 

## 6. Conclusions

Hexaric acids are sugar acids that can be produced from biomass. Due to their stereochemical diversity, chelation abilities, and high solubility in water, numerous applications have been developed. These applications include their use as chelating agents and corrosion inhibitors and as precursors for various polymers, coordination polymers, macromolecules, cross-linkers in hydrogels, and medicines. The conventional production of hexaric acids is conducted by the oxidation of saccharides with nitric acids. To overcome the problems derived from the use of nitric acids, in addition to improving the nitric acid oxidation process, green chemical methods have been developed to produce hexaric acids. Transition metal catalysts and modified microorganisms have been utilized to produce hexaric acids from hexuronic acids under mild conditions. Quite recently, the production of hexuronic acids using high-frequency ultrasound irradiation without a catalyst has been reported [140]. In addition, the production of hexuronic acids by using metal catalysts has been developed [141]. Combining these technologies with the aforementioned technologies, the production of hexaric acids can be improved. Furthermore, hexaric acid was produced directly from aldohexoses by using modified microorganisms. The oxidation reaction of aldohexoses using enzymes is considered to have an advantage in terms of stereoselectivity. In the future, processes for producing hexaric acids using a biocatalyst including enzymes could be constructed.

## Figures and Tables

**Table 1:** Isolated yields, melting points, and water solubility of poly(alkylene hexaramide)s [31]. The original tables have been combined into this table.

Polymers	Yield	mp (°C) ^a^	Water Solubility ^b^	*M_n_* ^c^
Poly(ethylene d-glucaramide)	93.3	185	Yes	2036
Poly(tetramethylene d-glucaramide)	88.3	192–195	Yes	2725
Poly(hexamethylene d-glucaramide)	89.4	192–194	No	2552
Poly(octamethylene d-glucaramide)	86.8	195–200	No	3562
Poly(decamethylene d-glucaramide)	89.8	200–205	No	3218
Poly(dodecamethylene d-glucaramide)	93.9	200–205	No	2730
Poly(ethylene galactaramide)	90.1	205	VS	702
Poly(tetramethylene galactaramide)	82.1	230	SS	1179
Poly(hexamethylene galactaramide)	85.2	228	NS	2320
Poly(octamethylene galactaramide)	79.4	230	NS	4920
Poly(decamethylene galactaramide)	82.2	230	NS	2560
Poly(dodecamethylene galactaramide)	87.4	225	NS	2356
Poly(ethylene d-mannaramide)	72	180–206	VS	1410
Poly(tetramethylene d-mannaramide)	77	194–201	VS	1249
Poly(hexamethylene d-mannaramide)	68	205–210	NS	1247

^a^ All polymers decomposed upon melting. mp = melting point. ^b^ VS = very soluble; SS = slightly soluble; NS = not soluble. ^c^ *M_n_* = number average molecular weight.

## Abbreviations

DOE	The US Department of Energy
PEIs	Polyethylene imines
MOF	Metal–organic framework
UDP	Uridine diphosphate
GDP	Guanosine diphosphate
UDH	Uronate dehydrogenase
PQQ	Pyrroloquinolone quinone
GDH	Glucose dehydrogenase
BOD	Bilirubin oxidase
DH_PDH_	PQQ domain of pyranose dehydrogenase from *Coprinopsis cinerea*
